# Life‐history correlations change under coinfection leading to higher pathogen load

**DOI:** 10.1002/evl3.48

**Published:** 2018-04-01

**Authors:** Anna‐Liisa Laine, Hannu Mäkinen

**Affiliations:** ^1^ Research Programme in Organismal & Evolutionary Biology University of Helsinki PO Box 65 (Viikinkaari 1) FI‐00014 Finland

**Keywords:** Disease biology, evolution of virulence, host genotype, host–pathogen interactions, life‐history theory, pathogen genotype, virulence

## Abstract

The ability of a parasite strain to establish and grow on its host may be drastically altered by simultaneous infection by other parasite strains. However, we still lack an understanding of how life‐history allocations may change under coinfection, although life‐history correlations are a critical mechanism restricting the evolutionary potential and epidemiological dynamics of pathogens. Here, we study how life‐history stages and their correlations change in the obligate fungal pathogen *Podosphaera plantaginis* under single infection and coinfection scenarios. We find increased pathogen loads under coinfection, but this is not explained by an enhanced performance at any of the life‐history stages that constitute infections. Instead, we show that under coinfection the correlation between timing of sporulation and final pathogen load becomes positive. The changes in pathogen life‐history allocations leading to more severe infections under coinfection can have far‐reaching epidemiological consequences, as well as implication for our understanding of the evolution of virulence.

**Impact Statement**

Understanding pathogen evolution is one of the major challenges in evolutionary biology. The two major theories that have been proposed to explain evolutionary trajectories in pathogens state that (1) pathogen life‐history allocations are a critical mechanism restricting the evolutionary potential and epidemiological dynamics of pathogens, and (2) the ability of a parasite strain to establish and grow on its host may be drastically altered by simultaneous infection by other parasite strains. Here, we provide the first empirical evidence of pathogen life‐history trade‐offs changing in single versus coinfection situations. Thus, our results considerably advance our understanding of how coinfection may impact on disease evolution and epidemiology. Our results are also of general interest in ecology and evolutionary biology as we demonstrate how context‐dependent life‐history trade‐offs are.

## Introduction

Throughout the growing season, as epidemics proceed, the same host individual may be challenged by more than one pathogen strain of the same species or different pathogen species. The outcome may range from rapid competitive exclusions, resulting in single strain infection (i.e., superinfection; Nowak and May 1994) to multiple infections with several pathogen genotypes coinfecting the same host individual. Given the often inconspicuous nature of the parasitic lifestyle and lack of morphological differentiation among strains, empirical studies of disease dynamics under coinfection have lagged behind theoretical predictions. However, with molecular tools becoming increasingly available for the study of parasites, we now know that coinfections are common in humans (Balmer and Tanner 2011), animals (Karvonen et al. 2012), and plants (Tollenaere et al. 2016).

Theory predicts that under diverse parasitic infections competition for limited host resources results in a “tragedy of the commons” situation where higher levels of host exploitation may emerge (Hamilton 1972). Hence, within‐host disease dynamics are proposed to change under coinfection, and this assumption is at the heart of many theoretical models, predicting increased pathogen load and even virulence—defined here as harm to host—when multiple strains simultaneously infect the same host (Mideo et al. 2008). As empirical evidence is accumulating, we now know that infection outcome may change considerably following coinfection compared to predictions based on single infection outcomes (de Roode et al. 2005a,b; López‐Villavicencio et al. 2007; Buckling and Brockhurst 2008; Karvonen et al. 2012; Susi et al. 2015a). Far less is known about which infection stages are most sensitive to the coinfection status of the host, and whether resource allocation between pathogen life‐history stages that typically constrain pathogen performance, change under coinfection. This is nontrivial as allocation strategies to different life‐history stages is considered the key mechanism restricting pathogen evolutionary potential, host range, and epidemiology (Futuyma and Moreno 1988, Thrall and Burdon 2003, Anderson et al. 2004, Fraile et al. 2011, Thrall et al. 2012, Susi and Laine 2013). Moreover, a precise knowledge of life‐history correlations, and how they may vary depending on conditions, is critical for understanding disease transmission, virulence, and ultimately for the control of infectious disease (Hochberg 1998, Hochberg and van Baalen 2000).

To understand how life‐history stages and their correlations change between single infection and coinfection, we carried out a laboratory experiment where strains of obligate fungal pathogen *Podosphaera plantaginis* were inoculated onto live leaves of *Plantago lanceolata*, and after a short time lag the same leaf is challenged either with the same strain, or by another strain. This pathogen completes its entire life cycle on the surface of the host plant, and hence we were able to monitor the infection stages visually. We developed a qPCR protocol to accurately and objectively quantify pathogen load in both single infection and coinfection scenarios. We find that coinfection by another strain typically changes infection outcome, and that the magnitude of the effect depends on the identity of the coinfecting strain. Moreover, we find that the negative correlation between sporulation day and final pathogen load becomes positive under competition with another strain.

## Materials and Methods

### HOST–PATHOGEN INTERACTION

We focused our study on the obligate fungal pathogen *P. plantaginis* (Castagne; U. Braun & S. Takamatsu) naturally infecting host plant *Plantago lanceolata*. This pathogen is a host‐specific obligate biotroph that completes its entire life cycle on the surface of the host plant where it is visible as localized (nonsystemic) white powdery lesions. Approximately 4000 *P. lanceolata* populations have been systematically mapped in the Åland Islands, southwest of Finland, since the 1990s, and their infection status by *P. plantaginis* has been annually surveyed since 2001. These data have demonstrated that regionally *P. plantaginis* persists as a highly dynamic metapopulation through extinctions and (re‐) colonizations of local host populations (Jousimo et al. 2014). The pathogen is a significant stress factor for its host, and may even cause host mortality (Laine 2004). The consequences of infection are visible also at the population level; host populations infected by the pathogen have lower growth rates than healthy host populations (Penczykowski et al. 2015).

The interaction between *P. lanceolata* and *P. plantaginis* functions in a two‐step manner typical of most plant–pathogen associations. First, as the pathogen attempts to infect a new host, the interaction is strain specific as the same host genotype expresses resistance against some pathogen strains (i.e., recognition) while being susceptible to others (i.e., non‐recognition) (Laine 2007). Once a *P. plantaginis* strain has successfully established, infection development is affected by both pathogen and host genotype (Laine 2007). The key life‐history traits that constitute the asexual transmission stage following successful establishment are time to germination (measured as the number of days it takes for a spore landing on a leaf to form the first hyphae); time to conidial spore production (measured as the number of days it takes for the established mycelium to begin sporulation), whether an infection sporulates (0/1; some infections only consist of mycelia); and overall pathogen load that is the result of both mycelial growth and spore production. For an epiphytic parasite such as *P. plantaginis*, a previously infected area of host tissue is not readily available for colonization for other spores. Hence, the spatial distribution of pathogen strains on the host surface can generate considerable competition between strains. Large‐scale sampling and subsequent genotyping have revealed that coinfections, where two or more strains of *P. plantaginis* simultaneously infect the same host, are common in the Åland metapopulation (Tollenaere et al. 2012). Coinfection has been shown to change infection dynamics, leading to more severe epidemics (Susi et al. 2015a).

### ESTIMATING PATHOGEN LOAD USING A QPCR PROTOCOL

We developed a qPCR protocol that allows an objective quantification of pathogen load by quantifying the amount of strain‐specific DNA in a sample. Because there was a risk that in the competition experiment (see next section) the developing lesions caused by different strains would merge, we developed genotype‐specific DNA qPCR primers. To identify genome positions containing diagnostic SNP variation between parasite genotypes, the transcriptome of 16 pooled field collected samples of *P. plantaginis* was sequenced using 454 technologies. The full details of the transcriptome sequencing are given in Tollenaere et al. (2012), but we provide a brief summary here. Altogether 549,411 sequence reads were generated using mRNA as starting material and assembled into 45,425 contigs. The contigs were then searched for positions with nonoverlapping polymorphisms (i.e., fixed differences) between the *P. plantaginis* strains. A candidate position was identified in the contig rep_c_707 (contig_id), which showed that strains were fixed as either A–T or G–C. This pattern of polymorphisms was confirmed by developing a SNP genotyping array and genotyping a large number of strains from *P. plantaginis* populations in the Åland Island. The details of this genotyping array are given in Tollenaere et al. (2012). Briefly, 27 SNP loci were genotyped in 380 infected leaf samples from 80 locations in the mildew metapopulation.

Our aim was to develop primers specific for the two strains in the competition experiment such that they would only amplify their target DNA (i.e., strain‐specific DNA) using the contig rep_c_707 as a starting point. This was achieved by designing primers containing the diagnostic position in their 3′ end. The sequence for the AT‐specific strain was 5′‐ACA TAA CCT ATG AGA TTT GAC GTG TA‐3′ and the GC specific 5′‐ACA TAA CCT GTG AGA TTT GAC GTG TG‐3′. The other primer in the reaction was 5′‐CGA CTG CAA AGC ATC TTG AA‐3′ for both specific primers. The qPCR conditions were as follows: initial denaturation of 10 min at 95°C, 40 cycles of denaturation for 30 sec at 95°C, annealing for 30 sec at 62°C, extension 30 sec at 72°C. The final extension was done at 95°C for 5 min. The qPCR reactions were carried out in 10 μL final volumes comprising of 2 μL of DNA, 5 pmol of each primer and 1* PCR master mix with SYBR green I (Bio‐Rad). All amplifications were conducted in CFX96 Real Time Detection System (Bio‐Rad) and using three technical replicates for each sample. The performance of the strain‐specific primers were evaluated by estimating the amplification efficiency (standard curve) using pure strain‐specific DNA as starting material. After the competition experiment, any visible powdery mildew at the original infection site (see *Competition experiment*) was scraped from the surface of the leaf, and the DNA was extracted according to a salt extraction protocol (Aljanabi and Martinez 1997). To take into account different amplification efficiencies of the two primer combinations, a dilution series using pure *P. plantaginis* DNA was used. The difference in the efficiencies was calculated using the PrimEff function in the MCMC.qPCR R‐package (Matz *et al*. 2013). The Ct values (i.e., the number of PCR cycles to detect the signal from the sample) were then transformed to molecule counts according to formula count = *E* ^(Cq1 – Cq)^, where *E* refers to amplification efficiency and Cq1 to number of cycles to detect single molecule in the reaction. Small Ct values are expected to indicate presence of a large number target molecules, whereas large Ct values indicate low number of target molecules. In practice, the Cq1 was assumed to be 37 cycles because this value has been shown to be robust for different genes (Matz et al. 2013). The conversion also corrects for the difference in amplification efficiencies (Matz et al. 2013). Using molecule counts instead of Ct values in general linear models helps in handling zero counts as well as dealing with variance when the number of target molecules in in PCR is low (Matz et al. 2013).

### COMPETITION EXPERIMENT

We used an inoculation approach to study how pathogen life‐history stages and infection outcomes are altered when the same host leaf is colonized by a different strain. We chose six strains originating from the Åland Islands (IDs 3, 10, 2288_10, 1062_10, 9066_1, and 4_6; characterized in Susi and Laine 2015). Originally the strains were isolated from samples collected in September 2010. The strains have been maintained in the laboratory by transferring conidia to fresh host leaves approximately every two weeks. The host genotypes used in maintenance are the same as those used in this experiment (see next). Using a set of 27 SNPs (Tollenaere et al. 2012), these strains have been shown to be genetically distinct. Three of the strains represented the AT‐genotype at the rep_c_707 contig (3, 1062_10 and 9066_1), and three strains represented the GC‐genotype at this contig (10, 2288_10 and 4_6). The inoculations were carried out on four host genotypes, allopatric to all strains, confirmed to be susceptible to these pathogen strains during prior inoculation experiments and maintenance work (IDs 1413_15, 1061_17, 1413_16, and 2220_M4).

In the experiment, we inoculated a detached *P. lanceolata* leaf with one of the six *P. plantaginis* strains by placing four conidial chains on the leaf (henceforth called the original infection). The leaves used in the inoculations were of similar size, originated from different clones of the same host genotype. After a time lag of 4 min that allowed spores to settle on the leaf, we inoculated the same leaf at a 4‐cm distance from the previous inoculation site again by placing four conidial chains on the leaf (henceforth called the challenging strain). Each strain was challenged by itself, as well as by three other strains representing a different genotype at the rep_c_707 contig (Fig. S1). Each inoculation combination was done on all four host genotypes, and each pathogen genotype × pathogen genotype × host genotype inoculation was replicated three times. Hence, the experiment consisted of 288 inoculations. We also had a treatment where the first inoculation was not challenged by a second strain, but these results are not reported here, as our focus is on measuring differences between challenging strains. To help locate the inoculation sites during monitoring of pathogen life‐history stages, the spores were placed inside circles (1 cm Ø) drawn with a fine permanent marker on the leaf. The leaves were kept on moist filter paper in a 9‐cm Petri dish in a growth chamber at +20°C and with a 16‐h light/8‐h dark photoperiod. Prior work has shown that infection outcome and its timing on detached leaves are similar to leaves still intact with the plant (Laine 2011). Every day from day four until day 12 post inoculation, infection development was observed under a dissecting microscope, and the level of infection was scaled from 0 to 4 as 0 = only mycelium, 1 = mycelium and conidia visible under microscope, 2 = mycelia visible by naked eye and sparse conidia visible under dissecting microscope, 3 = colonies smaller than 0.5 cm^2^ with abundant sporulation, and 4 = colonies larger than 0.5 cm^2^ with abundant sporulation. This scoring was used to infer the key life‐history events: Time to germination was measured from the first‐day mycelia were observed on the leaf surface, and time to sporulation was measured from the first‐day conidial spores were observed. We also measured whether developing lesions sporulated or not (0/1) depending on whether any spores were observed on the lesions by day 12.

### STATISTICAL ANALYSES

We used generalized linear mixed models (GLMMs) as implemented in the SAS 9.3 Glimmix to analyze how life‐history stages and pathogen load of the original infection change under competition was affected by whether the challenging strain was the same strain or a different strain. The dependent variables describing pathogen life‐history stages and their error distributions and link functions—as indicated inside brackets—were time to germination (Poisson distribution; log), sporulation (0/1; binomial distribution; logit), and time to sporulation (normal, identity). We used the corrected cycle threshold (*C*_t_) values (see “Estimating pathogen load using a qPCR protocol”) of the qPCR assay as a measure of pathogen load, analyzing separately the AT and GC primers. Following ln‐transformation the *C*_t_ values were normally distributed. In all models, as explanatory variables we had strain genotype, plant genotype, and whether the challenging strain was the same genotype or another mildew genotype (competitor: self vs. nonself). Genotype of the challenging strain, nested under the self‐ versus non‐self‐classification, was defined as a random effect, and replicates of the same strain × strain × plant genotype—combinations were identified as the target of repeated measures response complying a compound symmetry structure (Littell et al. 2006). When significant, interactions were retained in the model.

To understand whether the genotype of the non‐self‐challenging strain affected life‐history stages of the first arriving strain, we analyzed the same models as described above, but replaced the self versus non‐self‐variable with the genotype of the challenging strain. This analysis was only carried out for those trials that included non‐self‐strains.

To determine how pathogen life‐history correlations changed depending on whether the developing infection was challenged by the same strain or another strain, we analyzed as GLMMs all relationships between the life‐history traits of time to germination, ability to sporulate (0/1; proportion of infection that produced spores), time to sporulation, and pathogen load (as measured in the qPCR assay). Before analyses, data were averaged to obtain trait profiles for each strain that included measures of pathogen growth and sporulation. In all models, the classification of the challenging strain (self vs. nonself) was included as a fixed explanatory variable. We also included in the model genotype of the original strain as well as genotype of the host plant as fixed explanatory variables. The identity of the challenging strain, nested under the self‐ versus non‐self‐classification, was defined as a random effect. When significant, interactions were retained in the model. We used the Bonferroni correction to adjust for multiple comparisons in all models.

## Results

**Figure 1:**
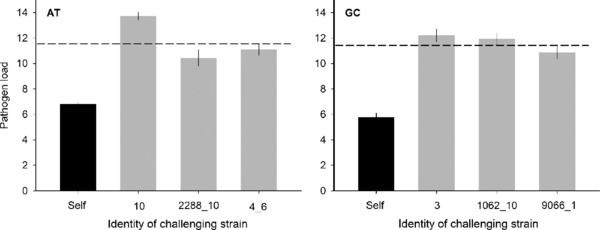
Pathogen load was higher in both AT and GC strains when the original infection was challenged with a different strain than with the same strain. Error bars are based on SE of mean, and the dashed line represents the average pathogen load when the original infection is challenged by non‐self‐strains.

When we compared the stages leading to infection—time to germinate, sporulation (0/1), and time to sporulation—none of the measured life‐history stages were significantly affected by whether the strain was challenged by itself or a different strain (Tables 1 and S1). Each life‐history stage was significantly affected by the genotype of the host plant (Tables 1 and S1), and time to sporulation differed significantly among the strains (*P* < 0.0001; Tables 1 and S1). Pathogen load was higher when strains were challenged by other strains than when they were challenged with themselves (AT: *P* = 0.0534, GC: *P* = 0.0061; Table 1 and Fig. 1). Pathogen load was also affected by the genotype of the first arriving strain as well as the genotype of the host plant (Tables 1 and S1).

**Table 1:** Results of the GLMMs analyzing life‐history stages of *Podosphaera plantaginis* as measured when challenged with same or different strain (self *vs*. nonself)

Source	*F*	*P*
Time to germination		
Competitor (self vs. nonself)_1, 9_	0.12	0.7384
Original strain genotype_5, 242_	1.24	0.2922
Host genotype_3, 242_	9.28	**<0.0001**
Sporulation (0/1)		
Competitor (self vs. nonself)_1, 9_	5.46	0.0442
Original strain genotype_5, 172_	1.19	0.3175
Host genotype_3, 172_	15.67	**<0.0001**
Time to sporulation		
Competitor (self vs. nonself)_1, 9_	0.01	0.9768
Original strain genotype_5, 209_	5.60	**<0.0001**
Host genotype_3, 209_	23.82	**<0.0001**
Pathogen load (AT strains)		
Competitor (self vs. nonself)_1, 4_	7.36	0.0534
Original strain genotype_2, 112_	3.27	0.0394
Host genotype_3, 112_	9.67	**0.0001**
Pathogen load (GC strains)		
Competitor (Self vs. nonself)_1, 4_	28.04	**0.0061**
Original strain genotype_2, 99_	96.39	**<0.0001**
Host genotype_3, 99_	16.84	**<0.0001**

We use the Bonferroni correction to adjust for multiple comparisons with statistically significant (*P* < 0.01) results shown in bold.

We found a significant genotype effect of the challenging strain when measuring time to sporulation (Table S3). Pathogen load depended not only on the identity of the original strain and host genotype, but also on the genotype of the challenging strain (AT: *P* < 0.0001, GC: *P* < 0.0001; Table S1). When measuring pathogen load of GC strains, we found a significant interaction between the original strain genotype and genotype of the challenging strain (*P* < 0.0001; Table S1).

**Figure 2:**
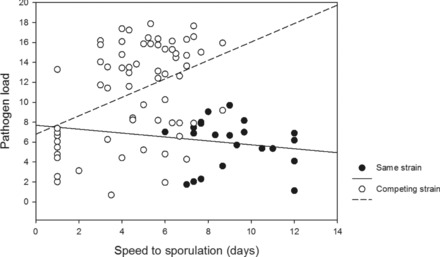
The correlation between time to sporulation and pathogen load of the tested pathogen strains became positive under coinfection. Time to sporulation is displayed here as speed with higher numbers indicating faster performance, and hence a positive correlation means that strains that begin to sporulate earlier, are also those that result in higher disease loads.

Time to sporulation was weakly negatively associated with pathogen load (*P* = 0.0313; Tables 2 and S2), but the shape of this correlation changed depending on whether the challenging strain was the same strain or a different strain (time to sporulation × competitor [self vs. nonself], *P* = 0.0015; Table 2 and Fig. 2). The association between time to sporulation and pathogen load became positive when the original infection was challenged by a different strain (Fig. 2).

**Table 2:** Results of the GLMMs analyzing correlations between different life‐history stages of *Podosphaera plantaginis* as measured when challenged with same or different strain

Source	*F*	*P*
Sporulation (0/1)		
Time to germination_1, 68_	0.14	0.7104
Competitor (self vs. nonself)_1, 9_	3.92	0.0789
Original strain genotype_5, 68_	1.45	0.2169
Host genotype_3, 68_	5.56	**0.0018**
Time to sporulation		
Time to germination_1, 68_	1.78	0.1867
Competitor (self vs. nonself)_1, 9_	0.01	0.961
Original strain genotype_5, 68_	3.88	**0.0037**
Host genotype_3, 68_	17.52	**<0.001**
Pathogen load		
Time to germination_1, 68_	1.42	0.2384
Competitor (self vs. nonself)_1, 9_	22.00	**0.0011**
Original strain genotype_5, 68_	13.34	**<0.001**
Host genotype_3, 68_	9.33	**<0.001**
Sporulation (0/1)_1, 68_	4.57	0.0361
Competitor (self vs. nonself)_1, 9_	23.26	**0.0009**
Original strain genotype_5, 68_	14.49	**<0.001**
Host genotype_3, 68_	6.59	**0.0006**
Time to sporulation_1, 67_	4.83	0.0313
Competitor (self vs. nonself)_1, 9_	20.02	**0.0015**
Original strain genotype_5, 67_	18.89	**<0.001**
Host genotype_3, 67_	7.83	**0.0001**
Time to sporulation × competitor (self vs. nonself)_1, 67_	9.35	**0.0032**

We use the Bonferroni correction to adjust for multiple comparisons with statistically significant (*P* < 0.01) results shown in bold.

## Discussion

Although coinfection is generally expected to change infection outcomes, a detailed understanding of which pathogen life‐history traits respond to coinfection—and how—has been lacking. Here, we show that although the life‐history stages leading to infection in *P. plantaginis* were not significantly affected by the presence of a competitor on the same leaf, pathogen load was higher under coinfection. Our study is the first to show that pathogen life‐history correlations change—becoming positive—under coinfection, with potentially far‐reaching evolutionary and epidemiological consequences.

Our results confirm the potential of coinfection to change infection outcomes. We found significantly higher pathogen loads when strains were challenged with a different strain than when challenged with themselves. This is in line with other studies that have found higher infection levels under coinfection than under single infection (de Roode et al. 2005b, López‐Villavicencio et al. 2007). This result also confirms previous findings in the interaction between *P. plantaginis* and *P. lanceolata* that show higher pathogen load of coinfected hosts, and more devastating epidemics under coinfection both in common garden conditions as well as in natural populations (Susi et al. 2015a). The change in epidemiological dynamics was explained by a change in transmission with more spores being released under coinfection than under single infection (Susi et al. 2015a, Susi et al. 2015b). However, to date we have not known how a developing lesion responds to the presence of a competing strain. Contrary to our a priori expectations, there was no evidence for life‐history stages leading to infection becoming faster or otherwise better under coinfection. Hence, the change in pathogen load cannot be explained by a change in any of the measured life‐history traits leading to infection (Table 1), although it is possible that small, statistically nonsignificant effects cumulate during infection development resulting in differences in pathogen load.

In our study, we find that pathogen life‐history trait correlations change under coinfection. Trade‐offs between life‐history traits, whereby allocation of limited resources to one trait has a negative impact on another trait is a generally accepted phenomenon in evolutionary biology (Stearns 1989). Trade‐offs in pathogens are of particular interest as they may play a crucial role in constraining the evolutionary potential, host range, and epidemiological dynamics of pathogens (Flor 1971; Thrall and Burdon 2003; Salvaudon et al. 2007; Héraudet et al. 2008; Woodhams et al. 2008; Barrett et al. 2011; Thrall et al. 2012; Laine and Barres 2013; Williams et al. 2014). We find that the weak negative correlation between time to sporulation and pathogen load becomes significantly positive under coinfection. In polycyclic pathogens such as *P. plantaginis*, where repeated cycles of asexual spore generations constitute the seasonal epidemics, the time it takes for sporulation to begin is considered a key fitness trait (Laine 2007, 2008). Under coinfection when the strains are competing for the same limited resources and space, a nonoptimal host resource exploitation strategy may be favored over a more moderate exploitation strategy (cf. Bull 1994). In general, the optimal reproduction rate is expected to differ between single and coinfection scenarios with parasites allocating resources to faster reproduction under coinfection (Mideo et al. 2008). This may be achieved through a change in the shape of trade‐offs that govern life histories, whereby a shift in conditions places emphasis on current reproduction over future reproduction (Edward and Chapman 2011).

Genetic variation of both hosts and the pathogens that infect them is well‐known to affect all aspects of infection development (Wolinska and King 2009, Laine et al. 2011, Tack et al. 2012). Our results confirm that both the genotype of the host and the genotype of pathogen often had a significant effect on the pathogen life‐history stages that we measured. However, interestingly we also found that how the developing infection responded to the presence of another strain depended on the genotype of the challenging strain, both directly and through genotype‐by‐genotype (G × G) interactions of the pathogen strains. Such G × G interactions can serve as a powerful mechanism maintaining diversity in parasite populations when no strain can outcompete all others as their fitness depends on the identity of the interaction partner. In other pathosystems, it has been demonstrated that kinship strongly shapes the interaction between coinfecting strains from cooperation to competitive exclusion (López‐Villavicencio et al. 2007, Buckling and Brockhurst 2008). Our data do not allow distinguishing the degree of relatedness of these strains, but this could be a putative determinant of the G × G specificity governing the response to coinfection. In *P. plantaginis* as in many other parasites, coinfection is also the prerequisite of sexual reproduction and hence, the probability of outcrossing with the coinfecting strain may change the outcome of coinfection (Carter et al. 2014). *Podosphaera plantaginis* has been shown to be capable of haploid selfing, but this does not exclude the possibility of outcrossing among strains (Tollenaere and Laine 2013).

In conclusion, our results add a new dimension to our current understanding of how context‐dependent the magnitude and shape of pathogen life‐history correlations may be (Vale et al. 2011, Susi and Laine 2013). In the *Plantago–Podosphaera* interaction, previous work has shown that increasing pathogen load impacts negatively on host growth and reproduction (Susi and Laine 2015), and hence, our results suggest that changes in life‐history correlations under coinfection may also impact virulence. If the conditions that generate variation in life‐history correlations are spatially and/or temporally variable, they may play a crucial role in generating heterogeneity in pathogen evolutionary trajectories. In this specific case of life‐history correlations changing under coinfection, we should also expect to see both pronounced differences in epidemiology depending on levels of coinfection, as has been shown in the *P. plantaginis* metapopulation (Susi et al. 2015a), as well as variation in selection for evolutionary strategies of this pathogen. Hence, understanding what factors generate variation in levels of coinfection, and understanding the extent to which life‐history variation may respond to diversity of infection, is a critical component of successful disease prevention efforts and offers a truly exciting venue of future research.

Associate Editor: K. Lythgoe

## Supporting information


**Figure S1**. **A schematic presentation of the inoculation experiment**.
**Table S1**. Solutions for fixed effects of GLMMs analyzing life‐history stages of *Podosphaera plantaginis* when challenged with same or different strain results as reported in Table.
**Table S2**. Solutions for fixed effects of GLMMs analyzing correlations between different life‐history stages of *Podosphaera plantaginis* when challenged with same or different strain results as reported in Table 2.
**Table S3**. Results of the GLMMs analyzing infection development of *Podosphaera plantaginis* as measured when challenged with a different strain. Statistically significant results are shown in bold.
