# Supplementary material for: Life‐history correlations change under coinfection leading to higher pathogen load
Source: Evol Lett. 2018 Apr 1;2(2):126–33. doi: 10.1002/evl3.48 (PMC6121793; doi:10.1002/evl3.48)
Supplement: Supplementary file 1 — Figure S1. A schematic presentation of the inoculation experiment. Table S1. Solutions for fixed effects of GLMMs analyzing life‐history stages of Podosphaera plantaginis when challenged with same or different strain results as reported in Table. Table S2. Solutions for fixed effects of GLMMs analyzing correlations between different life‐history stages of Podosphaera plantaginis when challenged with same or different strain results as reported in Table 2. Table S3. Results of the GLMMs analyzing infection development of Podosphaera plantaginis as measured when challenged with a different strain. Statistically significant results are shown in bold. [file EVL3-2-126-s001.pdf]

Laine & Mäkinen

Pathogen life-history correlations change under coinfection leading to higher pathogen load

SUPPORTING INFORMATION

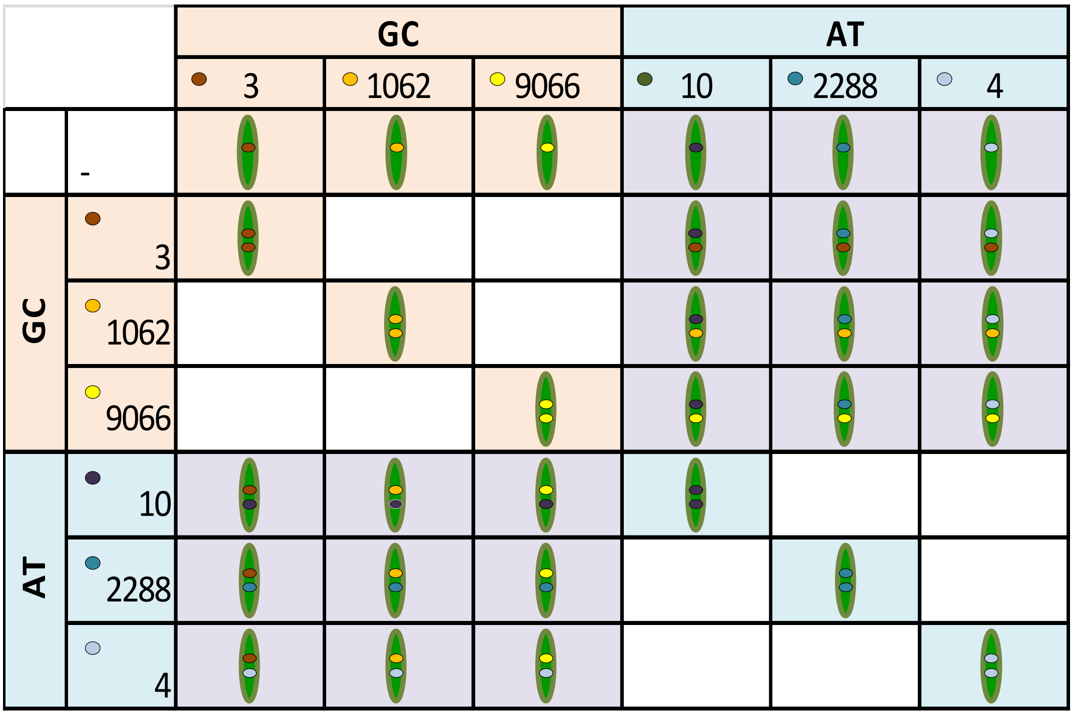

Figure S1. A schematic presentation of the inoculation experiment. The experiment was carried out with six strains of *Podosphaera plantaginis* that were either AT- or GC-genotype at rep_c_707 contig. Each strain was challenged with itself and the three strains representing the other genotype.

Supplementary Table 1. Solutions for fixed effects of GLMMs analyzing life-history stages of *Podosphaera plantaginis* when challenged with same or different strain results as reported in Table

Effect	Strain1	Plant	Allosym	Estimate	Standard Error	DF	t Value	Pr > |t|
Time to germination								
Intercept				1.4774	0.05942	9	24.87	<.0001
Strain1	1			-0.01748	0.07117	242	-0.25	0.8062
Strain1	2			-0.09572	0.07310	242	-1.31	0.1916
Strain1	3			0.02756	0.07172	242	0.38	0.7011
Strain1	4			-0.04948	0.07188	242	-0.69	0.4919
Strain1	5			0.06963	0.07060	242	0.99	0.3250
Strain1	6			0	.	.	.	.
Plant		1		-0.02090	0.04981	242	-0.42	0.6752
Plant		2		0.04036	0.04869	242	0.83	0.4080
Plant		3		0.2300	0.05203	242	4.42	<.0001
Plant		4		0	.	.	.	.
Allosym			1	-0.01695	0.04921	9	-0.34	0.7384
Allosym			2	0	.	.	.	.
Sporulation (0/1)								
Intercept				-0.1109	0.5116	9	-0.22	0.8332
Strain1	1			1.1302	0.8061	172	1.40	0.1627
Strain1	2			-0.3643	0.6948	172	-0.52	0.6008
Strain1	3			0.4929	0.7947	172	0.62	0.5359
Strain1	4			-0.6425	0.6882	172	-0.93	0.3518
Strain1	5			0.1798	0.7589	172	0.24	0.8130
Strain1	6			0	.	.	.	.
Allosym			1	1.5458	0.6614	9	2.34	0.0442
Allosym			2	0	.	.	.	.
Plant		1		3.3926	0.6716	172	5.05	<.0001
Plant		2		1.5351	0.3945	172	3.89	0.0001
Plant		3		0	.	.	.	.
Time to sporulation								
Intercept				8.6650	0.3918	9	22.11	<.0001
Strain1	1			-0.9691	0.3780	209	-2.56	0.0111
Strain1	2			-0.4806	0.4625	209	-1.04	0.2999
Strain1	3			0.2514	0.3902	209	0.64	0.5201
Strain1	4			-0.2504	0.4612	209	-0.54	0.5877
Strain1	5			0.9950	0.4557	209	2.18	0.0301
Strain1	6			0	.	.	.	.
Allosym			1	0.01006	0.3363	9	0.03	0.9768
Allosym			2	0	.	.	.	.
Plant		1		-1.1289	0.3317	209	-3.40	0.0008
Plant		2		0.2628	0.3408	209	0.77	0.4415
Plant		3		2.5019	0.4300	209	5.82	<.0001
Plant		4		0	.	.	.	.

Pathogen load (AT strains)								
Intercept				12.4365	1.3581	4	9.16	0.0008
Strain1	2			2.2373	0.9604	112	2.33	0.0203
Strain1	4			0.3194	0.9823	112	0.33	0.7452
Strain1	5			0	.	.	.	.
Plant		1		-1.1671	0.8872	112	-1.32	0.1890
Plant		2		-2.7186	0.9260	112	-2.94	0.0035
Plant		3		-5.9930	1.1686	112	-5.13	<.0001
Plant		4		0	.	.	.	.
Allosym			1	-4.5260	1.6686	4	-2.71	0.0534
Allosym			2	0	.	.	.	.
Pathogen load (GC strains)								
Intercept				15.5943	0.9321	4	16.73	<.0001
Strain1	1			-6.8920	0.6252	99	-11.02	<.0001
Strain1	3			1.2773	0.6500	99	1.97	0.0501
Strain1	6			0	.	.	.	.
Plant		1		-1.7428	0.5986	99	-2.91	0.0038
Plant		2		-3.2798	0.6197	99	-5.29	<.0001
Plant		3		-5.3525	0.8650	99	-6.19	<.0001
Plant		4		0	.	.	.	.
Allosym			1	-6.2951	1.1888	4	-5.30	0.0061
Allosym			2	0	.	.	.	.

Supplementary Table 2. Solutions for fixed effects of GLMMs analyzing correlations between different life-history stages of *Podosphaera plantaginis* when challenged with same or different strain results as reported in Table 2.

Effect	Strain1	Plant	sympallo	Estimate	Standard Error	DF	t Value	Pr > |t|
Sporulation (0/1) vs. Time to germination								
Intercept				1.0150	0.1192	9	8.51	<.0001
Strain1	1			0.05072	0.07056	68	0.72	0.4748
Strain1	2			-0.1057	0.07305	68	-1.45	0.1527
Strain1	3			0.01801	0.07562	68	0.24	0.8125
Strain1	4			-0.08839	0.07162	68	-1.23	0.2214
Strain1	5			0.000496	0.07163	68	0.01	0.9945
Strain1	6			0	.	.	.	.
Plant		1		-0.05072	0.05695	68	-0.89	0.3762
Plant		2		-0.1300	0.05671	68	-2.29	0.0249
Plant		3		-0.2633	0.06739	68	-3.91	0.0002
Plant		4		0	.	.	.	.
Germday				-0.00886	0.02376	68	-0.37	0.7104
sympallo			1	0.09455	0.04773	9	1.98	0.0789
sympallo			2	0	.	.	.	.
Time to sporulation vs. Time to germination								
Intercept				7.7688	0.8305	9	9.35	<.0001
Strain1	1			-1.0417	0.4920	68	-2.12	0.0379
Strain1	2			-0.6004	0.5534	68	-1.08	0.2818
Strain1	3			0.1491	0.5268	68	0.28	0.7780
Strain1	4			-0.3335	0.5450	68	-0.61	0.5426
Strain1	5			1.0031	0.5450	68	1.84	0.0701
Strain1	6			0	.	.	.	.
Plant		1		-0.9862	0.3842	68	-2.57	0.0125
Plant		2		0.3191	0.3825	68	0.83	0.4071
Plant		3		2.4127	0.4565	68	5.28	<.0001
Plant		4		0	.	.	.	.
Germday				0.2167	0.1625	68	1.33	0.1867
sympallo			1	0.01949	0.3864	9	0.05	0.9609
sympallo			2	0	.	.	.	.
Pathogen load vs. Time to germination								
Effect	Strain1	Plant	sympallo	Estimate	Standard Error	DF	t Value	Pr > |t|
Intercept				16.7973	1.8632	9	9.02	<.0001
Strain1	1			-6.6814	1.0837	68	-6.17	<.0001
Strain1	2			0.1885	1.3886	68	0.14	0.8924
Strain1	3			1.1952	1.1606	68	1.03	0.3067
Strain1	4			-1.8751	1.3748	68	-1.36	0.1771
Strain1	5			-2.5358	1.3739	68	-1.85	0.0693
Strain1	6			0	.	.	.	.
Plant		1		-1.5983	0.8128	68	-1.97	0.0533
Plant		2		-3.1178	0.8092	68	-3.85	0.0003
Plant		3		-4.7306	0.9689	68	-4.88	<.0001
Plant		4		0	.	.	.	.
Germday				-0.4153	0.3491	68	-1.19	0.2384
sympallo			1	-4.9910	1.0640	9	-4.69	0.0011
sympallo			2	0	.	.	.	.

Effect	Strain1	Plant	sympallo	Estimate	Standard Error	DF	t Value	Pr > |t|
Pathogen load vs. Sporulation (0/1)								
Intercept				11.6845	1.9772	9	5.91	0.0002
Strain1	1			-6.9333	1.0618	68	-6.53	<.0001
Strain1	2			0.5482	1.4122	68	0.39	0.6991
Strain1	3			0.8400	1.1140	68	0.75	0.4534
Strain1	4			-1.7854	1.3933	68	-1.28	0.2044
Strain1	5			-2.7199	1.3922	68	-1.95	0.0549
Strain1	6			0	.	.	.	.
Plant		1		-1.3217	0.7912	68	-1.67	0.0994
Plant		2		-2.7117	0.8162	68	-3.32	0.0014
Plant		3		-4.1440	1.0017	68	-4.14	<.0001
Plant		4		0	.	.	.	.
Sporu01				3.5158	1.6441	68	2.14	0.0361
sympallo			1	-5.3357	1.1062	9	-4.82	0.0009
sympallo			2	0	.	.	.	.
Pathogen load vs. Time to sporulation								
Intercept				24.3681	2.2264	9	10.95	<.0001
Strain1	1			-7.4996	0.9785	67	-7.66	<.0001
Strain1	2			-0.3098	1.2006	67	-0.26	0.7972
Strain1	3			1.2979	1.0062	67	1.29	0.2015
Strain1	4			-2.0138	1.1824	67	-1.70	0.0932
Strain1	5			-1.3616	1.2036	67	-1.13	0.2620
Strain1	6			0	.	.	.	.
Plant		1		-2.5835	0.7562	67	-3.42	0.0011
Plant		2		-2.9520	0.7228	67	-4.08	0.0001
Plant		3		-3.4823	1.0145	67	-3.43	0.0010
Plant		4		0	.	.	.	.
Sporuday				-1.0628	0.2280	67	-4.66	<.0001
sympallo			1	-14.5615	3.2543	9	-4.47	0.0015
sympallo			2	0	.	.	.	.
Sporuday*sympallo				1	1.0723	0.3507	67	3.06
	0.0032							
Sporuday*sympallo				2	0	.	.	.
	.							

Table S3. Results of the GLMMs analyzing infection development of *Podosphaera plantaginis* as measured when challenged with a different strain. Statistically significant results are shown in bold.

**Source**	***F***	***P***
Time to germination		
Original strain genotype$_{5, 61}$	1.61	0.3406
Competitor strain genotype$_{5, 61}$	0.37	0.8663
Host genotype$_{3, 186}$	9.46	**<0.0001**
Sporulation (0/1)		
Original strain genotype$_{5, 61}$	0.92	0. 4746
Competitor strain genotype $_{5, 61}$	1.54	0.1901
Host genotype$_{3, 185}$	12.87	**<0.0001**
Time to sporulation		
Original strain genotype$_{5, 61}$	5.39	**0.0004**
Competitor strain genotype $_{5, 61}$	3.37	0.096
Host genotype$_{3, 153}$	24.19	**<0.0001**
Pathogen load (AT strains)		
Original strain genotype$_{2, 102}$	12.13	**<0.0001**
Competitor strain genotype $_{5, 102}$	27.48	**<0.0001**
Host genotype$_{3, 102}$	20.74	**<0.0001**
Original strain genotype × Competitor strain$_{4, 102}$	29.71	**<0.0001**
Pathogen load (GC strains)		
Original strain genotype$_{2, 96}$	95.17	**<0.0001**
Competitor strain genotype $_{5, 96}$	30.14	**<0.0001**
Host genotype$_{3, 96}$	17.14	**<0.0001**
